# Polysaccharide Hydrogel-Based Fertilizer Carriers: Soil-Relevant Evaluation of Nutrient Release Beyond Conventional Aqueous Testing

**DOI:** 10.3390/gels12060497

**Published:** 2026-06-03

**Authors:** Babar Azeem, KuZilati KuShaari

**Affiliations:** 1 Department of Chemical Engineering, College of Engineering, Imam Mohammad Ibn Saud Islamic University (IMSIU), Riyadh 11564, Saudi Arabia; 2 Chemical Engineering Department, Prince Mohammad Bin Fahd University, Al Khobar 34754, Saudi Arabia; kkushaari@pmu.edu.sa

**Keywords:** polysaccharide hydrogels, controlled-release fertilizers, nutrient transport, swelling behavior, soil–fertilizer interactions, diffusion mechanisms

## Abstract

Polysaccharide hydrogel-based fertilizer carriers have emerged as promising alternatives to conventional synthetic systems due to their biodegradability, tunable physicochemical properties, and ability to regulate nutrient release through structure–transport interactions. However, their performance is still predominantly evaluated using simplified aqueous testing methods that fail to capture the complexity of real soil environments. This review provides an engineering-oriented analysis of nutrient release behavior from polysaccharide-based hydrogel systems, emphasizing the limitations of conventional aqueous evaluation and their implications for predicting field performance. The discussion integrates material design, transport phenomena, and environmental interactions to establish structure–property–release relationships governing nutrient delivery. Conventional aqueous testing methods are critically examined in terms of experimental configuration, performance metrics, and kinetic modeling approaches, highlighting their tendency to overestimate swelling, neglect ionic and biological interactions, and ignore external transport resistances. The influence of soil-dependent factors, including moisture dynamics, pH, ionic strength, microbial activity, and soil structure, is systematically analyzed to demonstrate their coupled effects on swelling, diffusion, and degradation-controlled release mechanisms. Comparative evidence reveals a consistent laboratory–soil mismatch, where aqueous systems predict faster release rates and shorter durations compared to soil conditions. Based on these insights, key gaps in current evaluation practices are identified, particularly the lack of soil-representative testing protocols and the limited applicability of models derived from aqueous systems. Finally, an engineering framework is proposed for soil-relevant evaluation and improved predictive modeling, aimed at supporting the rational design and scalable implementation of next-generation hydrogel-based fertilizer carriers.

## 1. Introduction

Ensuring global food security under increasing environmental and resource constraints remains one of the most critical challenges of the 21st century. The global population is projected to approach nearly 10 billion by 2050, placing unprecedented pressure on agricultural systems to enhance productivity while maintaining sustainability [1]. Fertilizers, particularly nitrogen-based ones such as urea, have played a central role in increasing crop yields due to their high nutrient content and economic viability [2,3]. However, conventional fertilizers suffer from inherently low nutrient use efficiency (NUE), as a significant fraction of applied nutrients is lost through leaching, volatilization, and runoff before plant uptake [2,4]. These losses not only reduce fertilizer effectiveness but also contribute to severe environmental issues, including groundwater contamination, eutrophication, and greenhouse gas emissions [5,6].

To address these limitations, controlled-release fertilizers (CRFs) have emerged as an advanced nutrient delivery strategy capable of synchronizing nutrient release with plant demand. By encapsulating nutrients within coating materials or polymeric matrices, CRFs prolong nutrient availability, enhance NUE, and minimize environmental losses [7,8,9]. Nevertheless, conventional CRFs, particularly those based on synthetic polymers, introduce new concerns related to their persistence in soil, potential accumulation, and long-term environmental impact. These challenges have driven growing interest in biodegradable and bio-based alternatives that can deliver nutrients efficiently while ensuring environmental compatibility [9,10].

Among emerging solutions, hydrogels and superabsorbent polymer systems have attracted significant attention due to their unique ability to simultaneously retain water and regulate nutrient release. Hydrogels are three-dimensional crosslinked polymer networks capable of absorbing large quantities of water and releasing encapsulated nutrients in a controlled manner [11,12,13]. Their dual functionality, water retention and controlled nutrient delivery makes them particularly suitable for improving agricultural productivity under water-scarce and stress-prone conditions. Furthermore, hydrogel-based systems have been shown to enhance soil structure, reduce irrigation demand, and mitigate nutrient losses, thereby contributing to more sustainable agricultural practices [11,14,15].

Recent research has increasingly focused on bio-based and polysaccharide-derived hydrogels, including materials such as starch, cellulose, chitosan, and alginate, owing to their biodegradability, renewability, and environmental safety [16,17,18,19,20]. These natural polymers offer tunable physicochemical properties and can be engineered to achieve desired swelling behavior, mechanical strength, and nutrient release kinetics. In particular, bio-inspired superabsorbent hydrogels have demonstrated the potential to significantly reduce fertilizer losses, reported to be as high as 70% for nitrogen, while improving crop productivity through sustained nutrient availability [17,21,22].

Despite these advancements, existing literature primarily focuses on general hydrogel applications or material development, with limited emphasis on establishing clear relationships between polysaccharide chemistry, hydrogel architecture, and nutrient release behavior under realistic soil conditions. In addition, challenges related to mechanical stability, degradation behavior in complex environments, and cost-effective large-scale production continue to hinder the practical implementation of these systems [17,19,23]. A comprehensive understanding that integrates material design with transport phenomena and release mechanisms is therefore still lacking.

In this context, this review aims to critically examine the development of polysaccharide-based hydrogel systems for controlled-release fertilizers, with a particular focus on their structure–property–release relationships and their relevance to practical fertilizer coating technologies. Particular attention is given to diffusion-controlled release mechanisms, hydrogel–soil interactions, and design principles that can bridge the gap between laboratory-scale materials and scalable agricultural applications.

**Figure 1:**
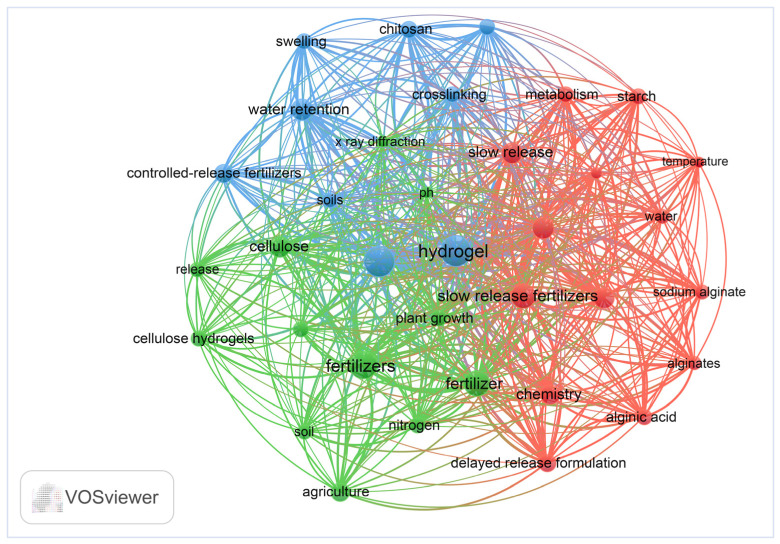
Keyword co-occurrence network of polysaccharide-based hydrogel systems for controlled-release fertilizers, showing major clusters related to hydrogel properties, fertilizer formulation, and soil–environment interactions.

To provide a structured overview of research trends in polysaccharide-based hydrogel systems for controlled-release fertilizers, relevant literature was collected from major scientific databases, including Scopus, Web of Science, and Google Scholar. The search was intentionally restricted to agriculture- and fertilizer-related applications using terms such as “polysaccharide hydrogels”, “controlled-release fertilizers”, “slow-release fertilizers”, “nutrient release kinetics”, and “soil”. This restriction was necessary because polysaccharide hydrogels are also widely used in biomedical, pharmaceutical, environmental, and water-treatment applications, which are outside the scope of the present review. The keywords shown in the co-occurrence map were therefore not arbitrarily generated by the visualization software; rather, they were extracted from the bibliographic records of the selected publications, including author keywords and indexed keywords, and then analyzed using VOSviewer (Version 1.6.20). As shown in Figure 1, the network indicates three major interconnected research domains: hydrogel material properties such as swelling, crosslinking, and water retention; fertilizer-related terms such as controlled-release fertilizers, nitrogen, and slow-release fertilizers; and soil–environmental terms such as soil, agriculture, and plant growth. This clustering supports the central focus of the review, namely the connection between polysaccharide hydrogel structure, fertilizer formulation, and soil-relevant nutrient release behavior.

**Figure 2:**
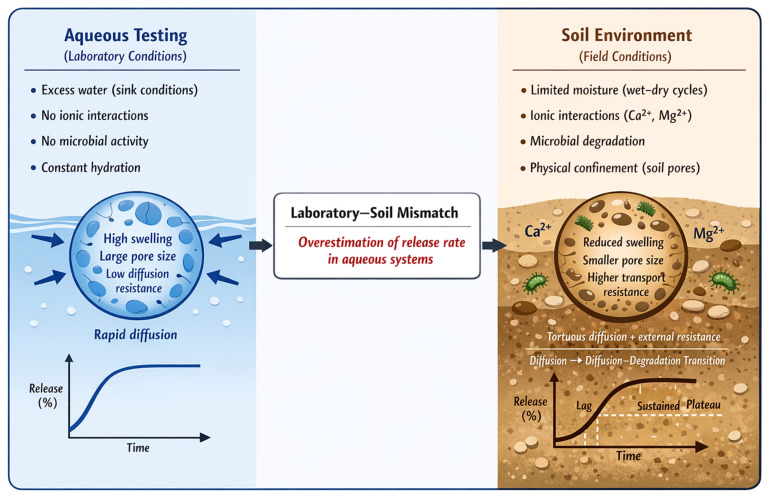
Comparison of nutrient release from polysaccharide-based fertilizer carriers under aqueous (laboratory) and soil (field) conditions. Aqueous systems promote excessive swelling, larger pore size, and low diffusion resistance, leading to rapid release, whereas soil environments, with limited moisture, ionic interactions (Ca^2+^, Mg^2+^), microbial activity, and physical confinement, reduce swelling, increase transport resistance, and result in slower, multi-stage diffusion–degradation-controlled release.

By integrating advances in polymer science, mass transfer, and agricultural engineering, this work seeks to provide a coherent framework for the rational design of next-generation sustainable fertilizer systems. The fundamental differences between conventional aqueous testing conditions and real soil environments, and their implications for nutrient release behavior in polysaccharide-based fertilizer systems, are schematically illustrated in Figure 2.

## 2. Polysaccharide-Based Fertilizer Carriers: Material Considerations

Building on the material limitations of conventional fertilizer carriers discussed earlier, increasing attention has been directed toward polysaccharide-based systems that inherently combine environmental compatibility with tunable functional performance. These natural polymers offer a chemically rich framework, dominated by hydroxyl (–OH), amino (–NH_2_), and carboxyl (–COO^−^) groups, that enables strong interactions with water, nutrients, and soil constituents. Such functionality provides a foundation for designing carriers where nutrient release is governed by intrinsic material properties rather than solely by external coatings [23,24,25].

A diverse range of polysaccharides has been investigated for fertilizer delivery applications, each exhibiting distinct structural characteristics. Starch is widely utilized due to its ease of modification and broad availability; however, its economic and sustainability advantages depend strongly on the source of starch [20,23]. The use of food-grade starch for large-scale fertilizer production may be uneconomical and may create competition with food resources. Therefore, non-food, industrial-grade, agricultural by-product-derived, or waste-biomass-based polysaccharide sources are more appropriate for scalable fertilizer carrier development [23,25]. Nevertheless, native starch-based systems often suffer from poor mechanical strength and rapid swelling [20,25]. In contrast, chitosan, a cationic polysaccharide, enables electrostatic interactions with anionic nutrients and soil particles, enhancing nutrient retention. Alginate, an anionic polymer, readily forms hydrogels through ionic crosslinking with multivalent cations, resulting in well-defined network structures with controllable porosity. Cellulose and its derivatives contribute superior mechanical stability and structural integrity, whereas pectin and natural gums provide additional gel-forming ability and environmental responsiveness [16,17,26]. These differences highlight that the selection of a specific polysaccharide is not arbitrary but directly linked to the desired balance between swelling behavior, mechanical robustness, and interaction with nutrients.

The performance of these materials is fundamentally governed by their physicochemical properties, particularly hydrophilicity, biodegradability, and sensitivity to environmental conditions. The hydrophilic nature of polysaccharides enables extensive water uptake, leading to swelling and the formation of hydrated networks that define nutrient diffusion pathways. In such systems, nutrient release is predominantly controlled by diffusion through the swollen matrix, where the effective diffusion coefficient is strongly dependent on network structure and water content [27,28,29]. Biodegradability, driven by microbial activity in soil, ensures environmental compatibility but introduces a critical trade-off: while gradual degradation can support sustained nutrient release, rapid microbial breakdown may lead to premature loss of structural integrity and uncontrolled release. Furthermore, many polysaccharides exhibit pronounced sensitivity to pH and ionic strength. Variations in soil pH or the presence of multivalent ions can alter polymer chain interactions, influencing swelling behavior, network stability, and ultimately nutrient release kinetics [30,31,32].

To address inherent limitations and enhance functional performance, several modification strategies have been developed. Crosslinking remains the most widely employed approach, where ionic crosslinking (e.g., alginate–Ca^2+^ systems) produces reversible and environmentally responsive networks, while covalent crosslinking generates more stable structures with reduced swelling and smaller mesh sizes. This reduction in network mesh size directly restricts nutrient diffusion, enabling slower and more controlled release. Grafting and chemical functionalization introduce additional functional groups that can modify polymer–nutrient interactions or impart responsiveness to environmental stimuli. Moreover, the incorporation of hydrophobic domains or inorganic fillers increases structural rigidity and creates more tortuous diffusion pathways, further suppressing rapid nutrient release [33,34,35]. However, crosslinking should be optimized rather than maximized. Excessive crosslinking may produce an overly compact network, suppress water penetration, reduce useful swelling, and restrict nutrient transport to the extent that release pathways become partially blocked [25,33,35]. Therefore, the crosslinking degree must be controlled by regulating crosslinker concentration, reaction time, reaction temperature, and the balance between ionic and covalent junctions [33,35]. In practical formulation design, swelling ratio, gel fraction, mechanical stability, mesh size, and nutrient release rate should be evaluated together to identify an optimum crosslinking window. The desired structure is not a completely sealed coating or matrix, but a semi-permeable and mechanically stable network that maintains sufficient water uptake while imposing adequate resistance to nutrient diffusion [25,35]. These strategies collectively demonstrate that nutrient release behavior can be systematically tuned through deliberate manipulation of polymer architecture.

An important design challenge in polysaccharide hydrogel-based fertilizer carriers is balancing water retention with slow nutrient release [25,28]. Although strong swelling improves water-holding capacity, excessive swelling can enlarge the hydrogel mesh size, reduce diffusion resistance, and promote rapid nutrient loss [25,28]. Therefore, high swelling alone should not be considered an indicator of superior controlled-release performance. A balanced carrier design requires sufficient water uptake for soil moisture retention, while maintaining a compact and stable network that limits nutrient diffusion [25,33,35]. This balance can be achieved by optimizing crosslinking density, introducing moderate hydrophobic modification, incorporating inorganic fillers, or forming interpenetrating/hybrid networks [33,34,35]. These strategies reduce excessive network expansion and increase diffusion pathway tortuosity, thereby improving slow-release behavior without completely sacrificing water-retention functionality [25,28,34,35].

The combined influence of these properties and modification approaches ultimately defines the nutrient release profile of polysaccharide-based carriers. Swelling-induced diffusion governs the initial release phase, while crosslinking density and hydrophobic modifications regulate the rate of transport through the matrix. At longer timescales, biodegradation contributes to sustained release by gradually opening additional pathways for nutrient transport. However, it is important to note that many polysaccharide-based systems still struggle to meet strict controlled-release criteria, often exhibiting faster-than-desired initial release due to excessive swelling or insufficient structural stability [36,37,38]. This highlights the need for more precise control over structure–property relationships.

Polysaccharide-based fertilizer carriers represent a versatile and sustainable material platform in which nutrient release behavior is intrinsically linked to polymer chemistry, network structure, and environmental responsiveness. Advancing these systems requires a more deliberate integration of chemical design and process optimization to achieve consistent and predictable performance under field conditions.

## 3. Structural Basis of Polysaccharide Hydrogels and Their Role in Nutrient Release Behavior

Polysaccharide-based hydrogels derive their functionality from the intrinsic chemical architecture of their polymer chains, which governs water uptake, network formation, and ultimately nutrient transport behavior. Unlike conventional coating materials that act primarily as passive diffusion barriers, polysaccharide hydrogels form three-dimensional, crosslinked networks in which water absorption, polymer relaxation, and network structure collectively control nutrient release. In such systems, nutrient transport is not governed solely by external conditions but emerges from the dynamic interplay between molecular structure, network architecture, and environmental interactions [39].

At the molecular level, polysaccharides are characterized by a high density of polar functional groups, primarily hydroxyl (–OH), carboxyl (–COO^−^), and amino (–NH_2_) moieties. These groups impart strong hydrophilicity and enable extensive hydrogen bonding with water molecules, leading to significant swelling when exposed to aqueous environments. As water penetrates the polymer matrix, the hydrogel network expands, increasing pore size (or mesh size) and reducing resistance to mass transfer. The effective diffusion coefficient of encapsulated nutrients is therefore strongly dependent on the degree of swelling, which is, in turn, dictated by the availability and accessibility of these functional groups. Consequently, hydrogels rich in hydrophilic functionalities tend to exhibit rapid swelling and diffusion-dominated nutrient release under idealized aqueous conditions [24].

Despite this common basis, the structural diversity among different polysaccharides leads to markedly different hydrogel behaviors. Starch-based hydrogels, composed of amylose and branched amylopectin chains, exhibit high swelling capacity due to abundant hydroxyl groups and relatively flexible chain structures. However, their weak intermolecular interactions often result in poor mechanical stability and large mesh sizes, promoting rapid diffusion and short release durations. In contrast, cellulose possesses a highly ordered and rigid backbone stabilized by extensive intra- and intermolecular hydrogen bonding. This structural rigidity restricts chain mobility, limits swelling, and produces more compact networks with lower permeability, thereby reducing nutrient diffusion rates and enhancing structural integrity [39].

Chitosan introduces an additional level of functionality through its protonatable amino groups, which impart pH-responsive behavior. Under acidic conditions, protonation of –NH_2_ groups increases electrostatic repulsion between polymer chains, enhancing swelling and permeability. Conversely, under neutral or alkaline conditions, reduced protonation leads to network contraction and decreased diffusion. This responsiveness enables dynamic modulation of nutrient release depending on environmental pH. Similarly, alginate and κ-carrageenan, both containing negatively charged functional groups (carboxylate in alginate and sulfate in carrageenan), readily form ionically crosslinked hydrogels in the presence of multivalent cations such as Ca^2+^. These ionic crosslinks act as junction points within the network, reducing mesh size, enhancing structural stability, and significantly limiting nutrient diffusion [40]. The degree of crosslinking, and thus the release rate, can be tuned by controlling ion concentration and distribution [41].

Pectin, another anionic polysaccharide rich in carboxyl groups, exhibits gelation behavior similar to alginate, particularly in the presence of divalent cations. Its relatively flexible chain structure allows for moderate swelling, while ionic interactions provide additional control over network density and permeability. The combined presence of hydrophilic and ionizable groups across these polysaccharides highlights that hydrogel behavior is governed not by a single structural feature but by the balance between chain flexibility, functional group chemistry, and crosslinking interactions [42].

These structural characteristics collectively define the hydrogel network architecture, particularly mesh size, crosslinking density, and connectivity, which are the key parameters controlling nutrient transport. A loosely crosslinked network with large mesh size facilitates rapid diffusion and short release durations, whereas a densely crosslinked structure imposes significant resistance to mass transfer, resulting in slower and more sustained nutrient release. Importantly, these structural parameters are not static; they evolve dynamically in response to environmental conditions, making nutrient release behavior highly context-dependent. A summary of representative studies investigating polysaccharide-based, polysaccharide-derived, and polysaccharide-containing hydrogel/coating systems for controlled-release fertilizers under soil-relevant conditions, including their composition, swelling behavior, water retention characteristics, and release kinetics, is provided in Table 1. Hybrid entries were re-examined to distinguish pure polysaccharide hydrogel systems from polysaccharide-derived bio-based coating systems.

This structure–property relationship provides a direct mechanistic basis for the discrepancies between aqueous and soil-based release behavior discussed in earlier sections. In aqueous systems, excess water availability and the absence of ionic constraints allow hydrogels to achieve maximum swelling, leading to expanded networks with low diffusion resistance and predominantly diffusion-controlled release [11,12]. In contrast, soil environments impose multiple constraints that effectively modify the hydrogel structure in situ. The presence of multivalent cations enhances ionic crosslinking, reducing mesh size and suppressing swelling [35]. Limited moisture availability restricts network expansion, while physical confinement within soil pores further limits deformation. At the same time, microbial activity progressively degrades the polymer backbone, altering network connectivity and introducing time-dependent pathways for nutrient release [14].

As a result, nutrient transport in soil systems cannot be described by simple diffusion models derived from aqueous testing. Instead, it reflects a coupled mechanism in which swelling, diffusion, and degradation are simultaneously governed by the underlying polymer structure and its interaction with the surrounding environment [32]. The structural features of polysaccharide hydrogels therefore serve as the fundamental link between material design and real-world performance, explaining why systems that appear highly efficient under aqueous conditions may exhibit significantly different behavior in soil.

**Figure 3:**
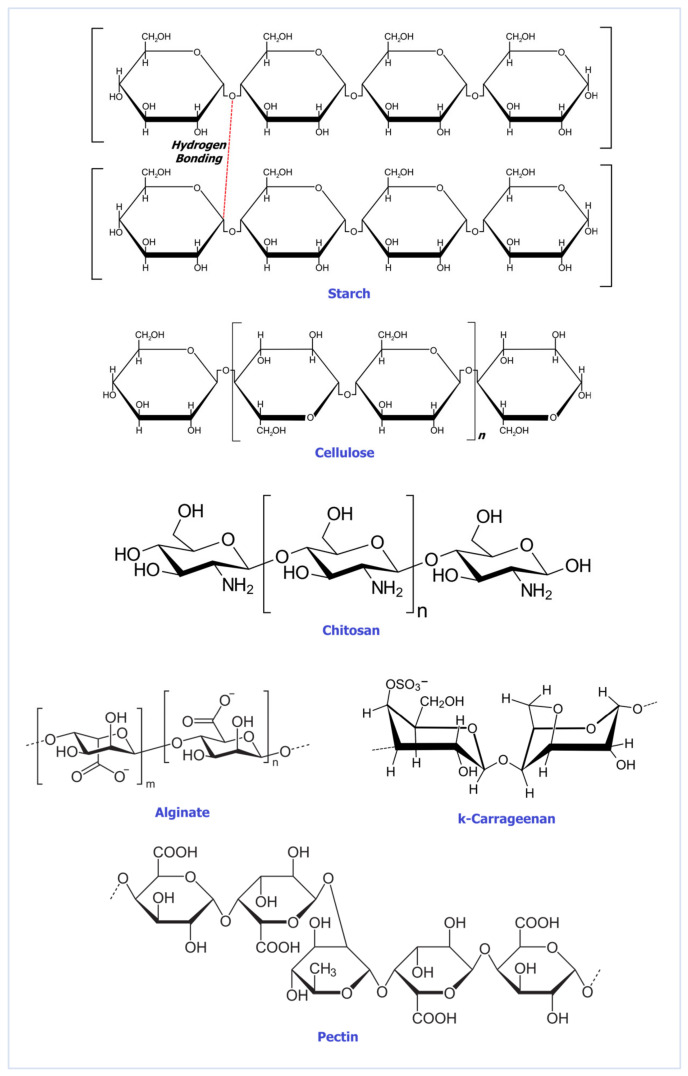
Chemical structures of representative polysaccharides used in hydrogel-based fertilizer systems (starch, cellulose, chitosan, alginate, κ-carrageenan, and pectin), highlighting functional groups responsible for swelling, crosslinking, and environmental responsiveness.

The representative chemical structures of commonly used polysaccharides, including starch, cellulose, chitosan, alginate, κ-carrageenan, and pectin, are illustrated in Figure 3. These structures highlight the functional groups and backbone architectures responsible for hydrogel formation, environmental responsiveness, and the resulting control over nutrient release behavior.

## 4. Conventional Aqueous Release Testing Methods

Following the discussion on how polysaccharide structure governs nutrient release, it is equally important to understand how this release is experimentally evaluated. In most studies, the performance of polysaccharide-based fertilizer carriers is assessed using simplified aqueous systems, which, although convenient, introduce several assumptions regarding real soil behavior [78,79,80].

**Figure 4:**
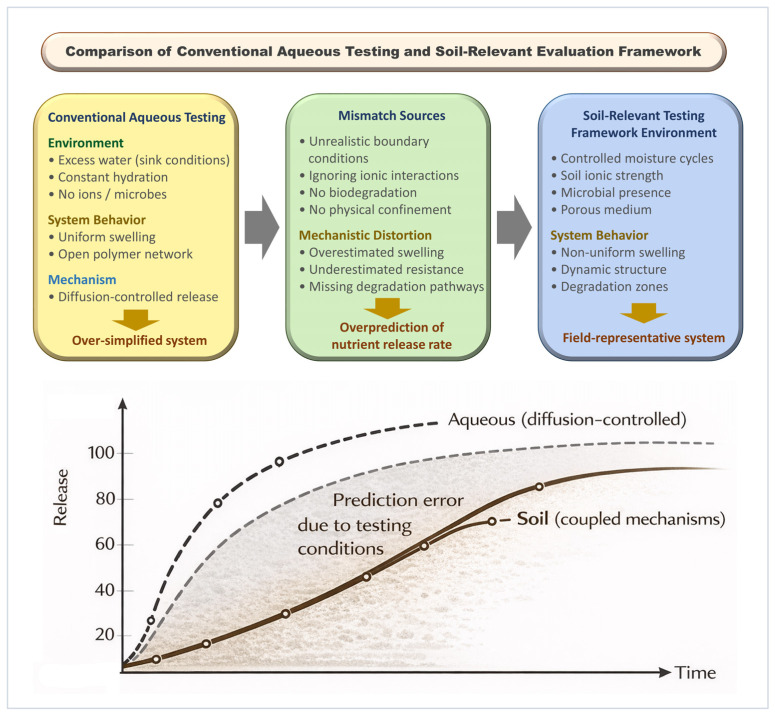
Schematic representation of conventional aqueous release testing methods, including static immersion and agitated systems. [These setups provide controlled conditions for evaluating swelling and diffusion-driven nutrient release but lack key soil-related factors such as ionic interactions, microbial activity, and physical confinement].

The most commonly employed experimental approach involves immersing fertilizer-loaded materials in distilled water or buffer solutions under static or dynamic conditions. In static immersion systems, samples are placed in a fixed volume of liquid without agitation, allowing nutrient release to occur solely through diffusion and swelling-driven mechanisms. In contrast, agitated systems, typically involving shaking or incubation, enhance mass transfer by reducing boundary layer resistance, leading to faster apparent release rates. These setups are widely adopted due to their simplicity and reproducibility, particularly in hydrogel-based systems where swelling and water uptake play a dominant role in nutrient transport [81,82,83]. Typical experimental configurations used in conventional aqueous release testing of polysaccharide-based fertilizer systems are illustrated in Figure 4.

Despite the apparent simplicity of these methods, testing conditions significantly influence the observed release behavior. Temperature is often maintained at constant values (commonly around ambient or physiological conditions) to ensure consistency; however, even small variations can alter polymer relaxation, swelling kinetics, and diffusion rates. The liquid-to-solid ratio is another critical parameter, as excess water creates sink conditions that accelerate nutrient dissolution and transport, potentially overestimating release rates compared to soil environments [84,85]. Similarly, sampling intervals determine the resolution of release profiles, particularly in capturing initial burst release or early-stage diffusion phenomena. Experimental studies frequently report release behavior under different pH or ionic conditions, demonstrating that changes in medium composition can substantially affect swelling and nutrient mobility [86,87,88].

Performance evaluation in aqueous testing is typically based on cumulative nutrient release as a function of time, expressed as a percentage of total nutrient content. While cumulative release provides an overall picture of delivery efficiency, additional parameters such as release duration and initial release rate are essential for interpreting system performance. For instance, many hydrogel systems exhibit a pronounced initial release phase followed by a slower, diffusion-controlled stage, reflecting the interplay between surface-associated nutrients and those entrapped within the polymer network [83,89,90]. Such multi-stage behavior highlights the importance of analyzing both the extent and rate of release rather than relying solely on cumulative values.

To further interpret release profiles, empirical and semi-empirical kinetic models are widely applied. Models such as Higuchi, Korsmeyer–Peppas, zero-order, and first-order kinetics are frequently used to fit experimental data and infer underlying transport mechanisms. The Higuchi model assumes diffusion-controlled release from a homogeneous matrix, while the Korsmeyer–Peppas model provides insight into whether the release follows Fickian or non-Fickian transport behavior. For example, studies on hydrogel-based fertilizer systems often report non-Fickian diffusion, indicating that both diffusion and polymer relaxation contribute to nutrient transport [91,92,93]. However, it is important to recognize that these models are primarily fitting tools and do not always represent the true physical mechanisms governing release.

A critical limitation of conventional aqueous testing lies in its underlying assumptions. These methods typically operate under excess water conditions, neglecting the complex interactions present in soil systems, such as adsorption onto soil particles, microbial activity, and variable moisture content. Furthermore, nutrient release is often assumed to be diffusion-controlled, whereas in real environments, additional processes such as biodegradation and chemical transformations may play significant roles [94,95,96]. As a result, aqueous testing tends to overestimate release rates and may not accurately predict field performance. This discrepancy underscores the need for more representative testing approaches that incorporate soil conditions and dynamic environmental factors.

While conventional aqueous release testing methods provide a useful and standardized framework for comparing materials, their inherent simplifications must be carefully considered. A deeper understanding of these limitations is essential for interpreting experimental data and for bridging the gap between laboratory evaluation and real-world agricultural performance.

## 5. Limitations of Aqueous Testing for Polysaccharide-Based Systems

Conventional aqueous release testing, although widely adopted due to its simplicity and reproducibility, presents several fundamental limitations when applied to polysaccharide-based fertilizer carriers. These limitations arise primarily from the mismatch between idealized laboratory conditions and the complex physicochemical and biological environment of real soils. As discussed in Section 4, aqueous systems are typically designed to isolate diffusion-controlled release; however, for polysaccharide-based matrices, where swelling, degradation, and environmental responsiveness dominate, the resulting data often overestimate performance and misrepresent field behavior.

### 5.1. Excessive Swelling in Pure Aqueous Media

One of the most significant limitations of aqueous testing is the exaggerated swelling behavior of hydrophilic polysaccharide matrices. Superabsorbent hydrogels, such as those based on starch, carrageenan, or guar gum, can exhibit extremely high swelling ratios under distilled water conditions due to the absence of ionic constraints and osmotic balancing effects. For instance, polysaccharide-based hydrogels may absorb water many times their own weight, forming highly expanded networks with increased pore size and reduced diffusion resistance [97,98,99].

This excessive swelling leads to accelerated nutrient diffusion and artificially shortened release durations. In contrast, soil environments impose physical confinement and capillary limitations that restrict matrix expansion [100]. As a result, aqueous testing tends to overestimate both water uptake and nutrient mobility, thereby failing to accurately capture the true release kinetics under field conditions.

### 5.2. Absence of Ionic Interactions

Aqueous systems, particularly those employing distilled water, lack the ionic complexity characteristic of soil solutions. In natural soils, multivalent cations such as Ca^2+^ and Mg^2+^ interact strongly with polysaccharide chains, especially in ionically crosslinked systems like alginate or pectin-based carriers [35,101]. These interactions can significantly reduce swelling, enhance structural stability, and alter diffusion pathways.

The absence of such ions in aqueous testing leads to an unrealistic representation of matrix behavior. As highlighted in controlled-release studies, nutrient release rates are strongly influenced by the surrounding medium, with significantly faster release observed in free water compared to soil or sand systems [83,102,103]. This discrepancy underscores the importance of ionic interactions in governing real-world release behavior, which is largely neglected in standard aqueous protocols.

### 5.3. Neglect of Microbial Activity

Another critical limitation is the complete absence of microbial activity in aqueous testing environments. In soil, polysaccharide-based carriers are inherently biodegradable and susceptible to enzymatic degradation by microorganisms. This biodegradation can progressively weaken the polymer network, increase porosity, and accelerate nutrient release over time [104,105].

In contrast, aqueous systems assume a static polymer structure, where release is governed primarily by diffusion or swelling-controlled mechanisms. This simplification neglects an important release pathway, biologically mediated matrix breakdown, which can play a dominant role in field conditions. The biodegradability of polymer coatings and matrices, widely recognized in fertilizer systems, therefore remains largely unaccounted for in aqueous testing, leading to underestimation of long-term release dynamics [80,106,107].

### 5.4. Constant Hydration Conditions

Standard aqueous release tests typically maintain constant immersion conditions, representing a fully saturated environment throughout the experiment. While this approach ensures experimental consistency, it fails to reflect the dynamic moisture conditions encountered in agricultural soils, where wetting–drying cycles are common due to irrigation patterns and climatic variability [103,108,109].

Polysaccharide-based materials are highly sensitive to such fluctuations. Repeated hydration and dehydration can induce structural fatigue, crack formation, and changes in swelling behavior, all of which influence nutrient release. Constant hydration conditions therefore provide an incomplete picture of release performance, often exaggerating sustained-release characteristics that may not persist under cyclic environmental conditions [25,28].

### 5.5. Lack of Soil–Matrix Interactions

Perhaps the most fundamental limitation of aqueous testing is the absence of soil–matrix interactions. In real soils, multiple processes, including adsorption of nutrients onto mineral surfaces, physical confinement within soil pores, and tortuous diffusion pathways, significantly influence nutrient transport [83,110].

Aqueous systems, being homogeneous and unconfined, eliminate these resistances and thus promote faster nutrient release. Experimental evidence shows that nutrient release in free water is consistently faster than in soil-like media, with differences becoming more pronounced under reduced moisture conditions. Furthermore, aqueous testing does not account for nutrient retention, leaching resistance, or interactions with soil organic matter, all of which are critical for evaluating fertilizer efficiency [103,111,112,113].

While aqueous testing provides a useful baseline for comparing formulations under controlled conditions, it inherently oversimplifies the behavior of polysaccharide-based fertilizer systems. The combined effects of excessive swelling, lack of ionic and biological interactions, and absence of soil constraints lead to systematic deviations from field performance. These limitations highlight the need for more representative testing methodologies that incorporate soil-like conditions, dynamic environments, and coupled physicochemical–biological processes.

## 6. Soil-Dependent Factors Governing Nutrient Release

Building upon the limitations of aqueous testing discussed in Section 5, it becomes evident that nutrient release from polysaccharide-based carriers is fundamentally governed by the complex and dynamic conditions of the soil environment. Unlike idealized aqueous systems, soils present a heterogeneous medium where physicochemical and biological factors interact simultaneously to control swelling, diffusion, and degradation processes. These factors collectively determine the actual release behavior and performance of polysaccharide-based fertilizer systems under field conditions.

### 6.1. Soil Moisture Dynamics

Soil moisture is inherently dynamic, characterized by intermittent wetting–drying cycles driven by irrigation practices, rainfall, and evaporation. Unlike continuous immersion in aqueous testing, these fluctuations impose cyclic swelling–deswelling behavior on polysaccharide matrices. During hydration phases, the polymer network absorbs water and expands, facilitating nutrient diffusion. However, subsequent drying leads to contraction, pore collapse, and potential structural rearrangement [114,115].

This cyclic behavior introduces a non-steady-state release mechanism, where diffusion pathways are repeatedly altered over time. In highly swellable systems such as polysaccharide hydrogels, repeated expansion and contraction may induce microstructural fatigue or cracking, further modifying release kinetics. Consequently, nutrient release in soil is not only diffusion-controlled but also strongly influenced by moisture history, making it inherently more complex than the steady release profiles observed under constant aqueous conditions [116,117,118].

### 6.2. Soil pH

Soil pH plays a critical role in determining the physicochemical stability and ionization state of polysaccharide-based carriers. Many polysaccharides, including alginate, pectin, and modified starch systems, contain functional groups (e.g., carboxyl and hydroxyl groups) that undergo protonation or deprotonation depending on pH. This directly affects electrostatic interactions within the polymer network, altering swelling behavior and permeability [119,120].

At higher pH values, increased ionization of functional groups leads to electrostatic repulsion between polymer chains, promoting network expansion and enhanced diffusion. Conversely, under acidic conditions, reduced ionization can result in network contraction and decreased permeability. Additionally, pH influences hydrolytic degradation pathways, particularly in chemically modified polysaccharides, thereby affecting long-term structural integrity and release behavior. As demonstrated in hydrogel-based fertilizer systems, nutrient release profiles can vary significantly across different pH environments, highlighting the importance of pH-responsive behavior in real soil conditions [29,30,121,122].

### 6.3. Ionic Strength and Salinity

The ionic composition of soil solutions exerts a profound influence on polysaccharide network structure and transport properties. Dissolved salts and multivalent cations such as Ca^2+^ and Mg^2+^ can interact with charged functional groups within the polymer matrix, effectively increasing crosslinking density and inducing network contraction. This phenomenon is particularly significant in ionically crosslinked systems, where cation bridging stabilizes the structure and reduces swelling capacity [115,123].

Increased ionic strength reduces osmotic pressure differences between the polymer and surrounding medium, thereby limiting water uptake and slowing nutrient diffusion. As a result, release rates in saline or ion-rich environments are often substantially lower than those observed in deionized water. The contrast between release behavior in free water and soil-like media, as reported in controlled-release fertilizer studies [124], underscores the critical role of ionic interactions in governing real-world performance.

### 6.4. Microbial Degradation

Polysaccharide-based carriers are inherently biodegradable, and their interaction with soil microbial communities introduces an additional, time-dependent release mechanism. Microorganisms secrete enzymes capable of cleaving glycosidic bonds within the polymer backbone, leading to progressive degradation of the matrix. This enzymatic activity increases porosity, weakens structural integrity, and facilitates the release of encapsulated nutrients [104,125].

Unlike aqueous systems where the polymer structure is assumed to remain intact, soil environments continuously modify the carrier through biological processes. The rate of microbial degradation depends on factors such as soil temperature, moisture, microbial population, and substrate composition [95,126]. As a result, nutrient release in soil often transitions from diffusion-controlled to degradation-assisted mechanisms over time, a behavior that cannot be captured by conventional aqueous testing.

### 6.5. Soil Texture and Structure

The physical structure of soil further constrains nutrient transport through mechanisms that are absent in homogeneous aqueous systems. Soil texture determines pore size distribution, while soil structure governs tortuosity and connectivity of diffusion pathways. In fine-textured soils (e.g., clay-rich systems), smaller pore sizes and higher tortuosity impose significant resistance to nutrient movement, leading to slower release and transport. In contrast, coarse-textured soils (e.g., sandy soils) offer less resistance but may enhance leaching losses [127,128,129].

Additionally, the physical confinement imposed by soil particles restricts the expansion of polysaccharide matrices, limiting swelling and modifying internal diffusion pathways. Adsorption of released nutrients onto soil minerals and organic matter further reduces their mobility and availability. Experimental comparisons have consistently shown that nutrient release is faster in free water than in soil or sand systems, particularly under unsaturated conditions [130,131,132], highlighting the dominant influence of soil physical constraints.

**Figure 5:**
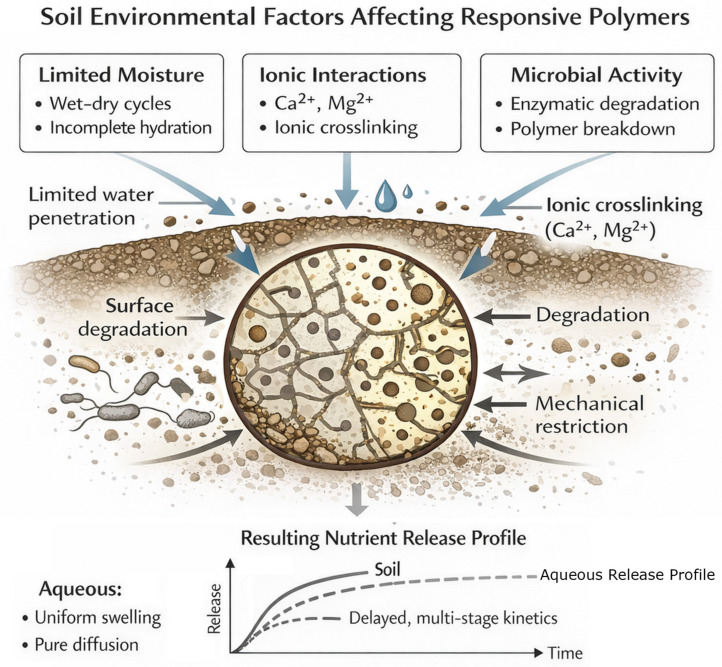
Soil environmental factors affecting nutrient release from polysaccharide-based fertilizer carriers. Limited moisture, ionic interactions (Ca^2+^, Mg^2+^), and microbial activity collectively reduce swelling, induce ionic crosslinking, promote degradation, and impose mechanical constraints, leading to restricted diffusion and delayed, multi-stage release compared to uniform diffusion in aqueous systems.

Taken together, these soil-dependent factors demonstrate that nutrient release from polysaccharide-based carriers is governed by a complex interplay of moisture dynamics, chemical environment, biological activity, and physical constraints. These interactions lead to release behaviors that are fundamentally different from those observed in simplified aqueous systems. Therefore, to accurately evaluate and optimize such materials, testing methodologies must evolve to incorporate soil-representative conditions and coupled environmental effects. The key soil environmental factors influencing the structure, swelling behavior, and nutrient release mechanisms of polysaccharide-based carriers are illustrated in Figure 5.

## 7. Mechanistic Differences Between Aqueous and Soil Systems

The preceding sections have established that nutrient release from polysaccharide-based carriers is governed by multiple mechanisms whose relative importance depends strongly on the surrounding environment. While aqueous systems are typically designed to isolate and quantify individual transport processes under controlled conditions, soil environments introduce additional physicochemical and biological complexities that fundamentally alter the dominant release pathways. As a result, the mechanisms governing nutrient release in aqueous media cannot be directly extrapolated to soil systems without careful reinterpretation.

### 7.1. Comparative Analysis of Dominant Release Mechanisms

In aqueous systems, nutrient release is most commonly described using simplified mechanistic frameworks, primarily based on swelling-controlled and diffusion-controlled processes. For highly hydrophilic polysaccharide matrices, water uptake leads to rapid swelling, enlargement of pore structures, and subsequent diffusion of dissolved nutrients through the hydrated network. This behavior is well captured by swelling-based and diffusion-based models frequently applied to hydrogel systems [83,133]. In such environments, the polymer structure is typically assumed to remain stable, and release kinetics are governed by relatively uniform concentration gradients.

In contrast, soil systems exhibit a more complex interplay of mechanisms. While swelling and diffusion remain relevant, their contributions are modulated by environmental constraints such as limited water availability, ionic interactions, and physical confinement. Moreover, degradation-controlled release becomes increasingly significant over time, particularly for biodegradable polysaccharide matrices [25,94,134]. Thus, whereas aqueous systems often reflect a single dominant mechanism, soil systems inherently involve multiple interacting mechanisms operating simultaneously.

### 7.2. Transition of Mechanisms from Aqueous to Soil Environments

A key distinction between aqueous and soil systems lies in the transition of dominant release mechanisms. In aqueous media, release is typically diffusion-dominated following rapid swelling, resulting in relatively predictable and smooth release profiles. However, in soil environments, this behavior evolves into a coupled process where diffusion, swelling, and degradation interact dynamically.

Experimental observations have demonstrated that nutrient release in free water is consistently faster than in soil-like media, with significant reductions in release rates under conditions of limited moisture or increased confinement [86,103]. This shift reflects the reduced swelling capacity and restricted diffusion pathways in soil, as well as the influence of external resistances. Over time, microbial degradation of the polysaccharide matrix further modifies the release mechanism, introducing a time-dependent component that is absent in purely aqueous systems.

**Figure 6:**
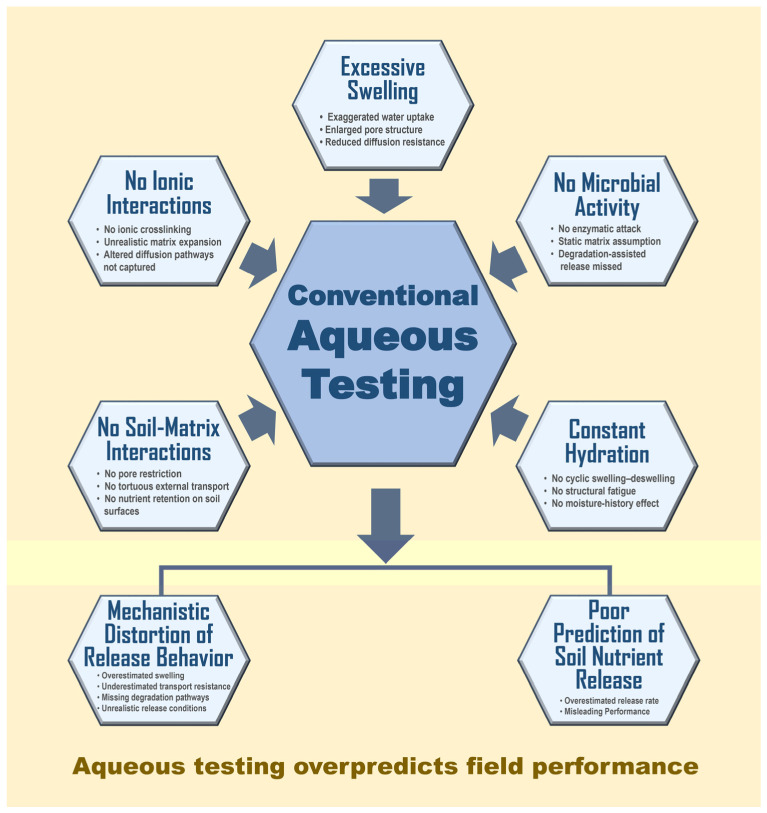
Mechanistic transition of nutrient release from polysaccharide-based carriers in aqueous and soil environments. Aqueous systems are dominated by swelling and diffusion, whereas soil conditions introduce additional constraints and microbial degradation, leading to multi-stage release behavior governed by coupled diffusion–degradation mechanisms.

Consequently, the release mechanism in soil can be more accurately described as a transition from diffusion-controlled behavior at early stages to a combined diffusion–degradation-controlled process at later stages. This transition highlights the inadequacy of applying single-mechanism models, developed for aqueous systems, to predict long-term release in soil environments. The transition of dominant nutrient release mechanisms from aqueous to soil environments, including the shift from diffusion-controlled to coupled diffusion–degradation behavior, is illustrated in Figure 6.

### 7.3. Interaction Between Transport Processes and Soil Conditions

In soil systems, transport processes are strongly influenced by environmental factors such as moisture content, pH, ionic strength, and microbial activity, as detailed in Section 6. These factors do not act independently but instead interact to modify the internal structure of the polymer matrix and the external transport resistance.

For example, reduced moisture availability limits swelling and decreases effective diffusivity, while increased ionic strength can induce network contraction through cation-mediated crosslinking. Similarly, variations in pH affect polymer ionization and, consequently, swelling behavior and permeability [135]. These effects collectively lead to non-linear and condition-dependent release kinetics that differ markedly from the relatively uniform behavior observed in aqueous systems.

Furthermore, the surrounding soil matrix imposes additional resistance to mass transfer through tortuous diffusion pathways and adsorption phenomena [136]. This results in a coupled internal–external transport system, where both the properties of the carrier and the characteristics of the soil medium jointly determine nutrient release.

### 7.4. Coupled Physicochemical–Biological Processes in Soil

Perhaps the most defining feature of soil-based systems is the coupling of physicochemical and biological processes. While aqueous testing isolates physical transport mechanisms, soil environments introduce microbial activity that actively alters the polymer structure. Enzymatic degradation of polysaccharides progressively increases porosity and weakens the matrix, thereby accelerating nutrient release over time [20,137].

This coupling leads to a feedback mechanism in which structural degradation enhances transport, and increased transport further exposes the matrix to microbial attack. As a result, release kinetics in soil are inherently time-dependent and cannot be adequately described by static models. The biodegradability of coating materials and polymer matrices, widely recognized in controlled-release fertilizer systems [80,106,138], thus plays a central role in determining long-term performance.

The mechanistic differences between aqueous and soil systems highlight a fundamental shift from simplified, single-process behavior to complex, multi-process interactions. Aqueous systems provide valuable insights into intrinsic material properties, but they fail to capture the coupled transport, environmental, and biological processes that dominate in soil [139]. Therefore, a comprehensive understanding of nutrient release from polysaccharide-based carriers requires an integrated mechanistic framework that accounts for these interactions, forming the basis for the development of more realistic testing methodologies and predictive models in subsequent studies.

## 8. Evidence of Laboratory–Soil Mismatch

The mechanistic differences outlined in Section 7 are not merely theoretical but are consistently supported by experimental observations reported in the literature. A growing body of evidence demonstrates that nutrient release profiles obtained under conventional aqueous testing conditions differ significantly from those observed in soil or soil-like environments. These discrepancies manifest in release rate, duration, and even the governing mechanisms, raising important concerns regarding the reliability of laboratory-based evaluations for predicting field performance.

### 8.1. Comparative Experimental Evidence

Direct comparisons between aqueous and soil-based systems clearly reveal systematic differences in nutrient release behavior. Studies examining polymer-coated fertilizers have shown that nutrient release in free water occurs more rapidly and uniformly, whereas release in soil or sand media is significantly slower and more heterogeneous. For instance, comparative experiments conducted in free water, saturated sand, and sand at field capacity demonstrated that release rates are consistently highest in aqueous systems and progressively reduced under soil-like conditions [9,140].

These findings highlight the strong influence of environmental resistance on nutrient transport. In aqueous media, the absence of physical confinement and external mass transfer limitations allows diffusion to proceed with minimal resistance. In contrast, soil systems impose constraints such as limited moisture availability, tortuous diffusion pathways, and interactions with soil particles, all of which collectively retard nutrient release [141,142].

### 8.2. Quantified Differences in Release Rate and Duration

Beyond qualitative differences, the mismatch between laboratory and soil systems is also reflected in measurable discrepancies in release kinetics. Aqueous testing often yields shorter release durations and higher apparent release rates due to enhanced swelling and unrestricted diffusion. In contrast, soil environments tend to exhibit delayed release, extended lag phases, and multi-stage release behavior [83,86,103].

For example, the release of different nutrients from coated fertilizers has been shown to follow distinct stages, including an initial lag phase, a constant-release phase, and a declining phase, with the duration and rate of each stage strongly dependent on the surrounding medium [143]. Importantly, these stages are more pronounced and prolonged under soil conditions, reflecting the combined effects of limited water availability and increased transport resistance. Such differences indicate that aqueous testing can significantly underestimate release duration and overestimate nutrient availability.

### 8.3. Variations in Dominant Release Mechanisms

Another critical aspect of laboratory–soil mismatch lies in the variation in dominant release mechanisms. In aqueous systems, release is predominantly governed by swelling and diffusion processes, as polymer matrices readily absorb water and establish stable concentration gradients. However, in soil environments, these mechanisms are altered by environmental constraints and additional processes [144,145].

Polysaccharide-based systems, for example, exhibit reduced swelling in ion-rich soil solutions due to cation-mediated interactions, which effectively increase crosslinking density and restrict network expansion. At the same time, microbial activity introduces degradation-controlled release pathways that are absent in aqueous testing [29,30,146]. As a result, the overall release mechanism in soil transitions from a relatively simple diffusion-controlled process to a coupled diffusion–degradation system, consistent with the mechanistic framework discussed earlier.

### 8.4. Case Studies Highlighting Misinterpretation of Performance

Several case examples further illustrate how reliance on aqueous testing can lead to misinterpretation of material performance. Polysaccharide-based hydrogels, known for their high swelling capacity and rapid nutrient release in distilled water, often demonstrate substantially reduced swelling and slower release under realistic soil conditions due to ionic effects and physical confinement [13,147]. Similarly, modified starch-based systems designed to control nutrient release through hydrophobic grafting exhibit tunable release behavior in aqueous media, yet their performance may differ in soil due to additional environmental interactions [148].

Moreover, biodegradable coatings that appear stable in short-term aqueous tests may undergo progressive degradation in soil, leading to accelerated nutrient release over time [9,80]. This behavior, which reflects the influence of microbial activity and environmental conditions, cannot be captured by static laboratory protocols. Consequently, aqueous testing may either overestimate sustained-release performance or fail to identify long-term degradation effects that are critical for field applications.

### 8.5. Implications for Predictive Modeling and Evaluation

The accumulated evidence clearly indicates that laboratory-derived release data, when based solely on aqueous testing, may not provide an accurate representation of real-world performance. Simplified models developed under such conditions often assume constant environmental parameters and single-mechanism control, limiting their applicability to soil systems where multiple processes interact dynamically [149].

These discrepancies underscore the need for caution when interpreting laboratory results and highlight the importance of incorporating soil-representative conditions into testing protocols. Without such considerations, there is a significant risk of overestimating fertilizer efficiency, misjudging release duration, and ultimately compromising agronomic performance. Therefore, bridging the gap between laboratory and field conditions requires not only improved experimental methodologies but also the development of integrated models that account for the coupled physicochemical and biological processes governing nutrient release in soil environments.

The consistent mismatch between laboratory and soil-based observations reinforces the limitations of conventional aqueous testing and the need for more representative evaluation strategies. Addressing this challenge requires the design of advanced testing methodologies that better simulate soil conditions and capture the dynamic interactions influencing nutrient release. The key experimental evidence and recurring observations underlying the laboratory–soil mismatch in nutrient release behavior are summarized in Table 2.

## 9. Critical Gaps in Current Evaluation Practices

The discrepancies between aqueous and soil-based release behavior discussed in Section 5, Section 6, Section 7 and Section 8 are not incidental but stem from fundamental limitations in current evaluation practices. Despite significant advances in the design of polysaccharide-based fertilizer carriers, the methodologies used to assess their performance remain largely simplified, inconsistent, and often disconnected from real soil conditions. These shortcomings not only hinder meaningful comparison across studies but also limit the predictive reliability of laboratory-derived results.

A primary limitation is the absence of standardized and soil-representative testing protocols. Most studies rely on static aqueous systems using distilled water or simple buffer solutions, with little consideration of key soil parameters such as fluctuating moisture content, ionic composition, or microbial activity. As a result, testing conditions vary widely across the literature, making it difficult to establish benchmarks for performance evaluation. Even when similar materials are investigated, differences in experimental setup can lead to substantially different release profiles, complicating interpretation and comparison.

Closely related to this issue is the inconsistent and often incomplete reporting of experimental conditions. Critical parameters such as pH, temperature, ionic strength, liquid-to-solid ratio, agitation intensity, and sampling intervals are frequently either omitted or insufficiently described. For polysaccharide-based systems, where swelling behavior, network stability, and diffusion characteristics are highly sensitive to environmental conditions, such omissions can significantly affect the reproducibility and reliability of results. Without clear documentation of these factors, it becomes challenging to distinguish between intrinsic material performance and artifacts arising from experimental conditions.

Another major gap lies in the continued over-reliance on simplified aqueous systems for performance evaluation. While these systems offer convenience and experimental control, they fail to capture the complexity of soil environments. The limited adoption of soil-based, soil-column, or dynamic wetting–drying testing approaches further exacerbates this issue. Consequently, release data obtained from aqueous testing often overestimate nutrient availability and underestimate release duration, as previously demonstrated. This reliance on simplified systems persists despite growing evidence that soil conditions fundamentally alter the governing release mechanisms.

In addition, current evaluation frameworks largely neglect the coupling of physicochemical and biological processes. Most testing methodologies are designed to isolate individual mechanisms, such as diffusion or swelling, without accounting for simultaneous interactions involving ion exchange, matrix restructuring, and microbial degradation. In real soil systems, these processes occur concurrently and influence each other dynamically. Ignoring this coupling not only oversimplifies the release behavior but also limits the development of mechanistically accurate models.

The lack of comparability and reproducibility across studies represents another critical challenge. Variations in material formulation, coating thickness, testing media, and analytical methods lead to a wide dispersion of reported results, even for similar systems. This variability hinders the establishment of generalized design principles and makes it difficult to identify truly effective material strategies. Moreover, the absence of standardized metrics and reporting formats further complicates cross-study analysis.

Finally, there exists a significant disconnect between laboratory performance metrics and actual agronomic outcomes. Parameters such as cumulative nutrient release percentage or release duration are commonly used as indicators of performance; however, they do not necessarily correlate with plant nutrient uptake efficiency or field productivity. This gap highlights the need to integrate agronomic relevance into evaluation frameworks, ensuring that laboratory testing provides meaningful insights into real-world performance.

Collectively, these gaps underscore the need for a paradigm shift in the evaluation of polysaccharide-based fertilizer carriers. Moving beyond simplified and isolated testing approaches toward more integrated, soil-representative methodologies is essential for accurately assessing material performance and guiding future material design.

## 10. Toward Soil-Relevant Evaluation and Standardization

The critical gaps identified in the previous section clearly indicate that current evaluation practices are insufficient to reliably predict the real-world performance of polysaccharide-based fertilizer carriers. Addressing these limitations requires a shift from simplified, isolated testing approaches toward standardized methodologies that better represent soil conditions. In this context, the development of soil-relevant evaluation strategies is not merely an improvement in experimental design but a necessary step toward bridging the persistent gap between laboratory findings and field performance.

### 10.1. Recommended Testing Approaches

A key direction for improvement lies in the adoption of testing systems that incorporate essential features of soil environments. Among these, soil slurry systems provide a practical intermediate approach, combining the ease of laboratory handling with the presence of soil particles and dissolved ions. Such systems enable the investigation of interactions between the fertilizer matrix and soil components while maintaining controlled experimental conditions.

Simulated soil solutions represent another valuable approach, particularly for isolating the effects of ionic composition and pH. By incorporating relevant concentrations of monovalent and multivalent ions, these systems can better capture ion–polymer interactions that significantly influence swelling, crosslinking, and nutrient diffusion. This is especially important for polysaccharide-based materials, whose structural behavior is highly sensitive to ionic environments.

Controlled moisture environments offer an additional level of realism by mimicking the intermittent hydration conditions typical of field soils. Unlike fully saturated aqueous systems, these setups allow for the study of wetting–drying cycles and their impact on matrix swelling, structural integrity, and release kinetics. Such dynamic conditions are critical for understanding how materials perform under realistic agricultural scenarios.

Together, these approaches provide a progressive pathway from simplified laboratory testing toward more representative soil conditions, enabling a more accurate assessment of nutrient release behavior.

### 10.2. Key Parameters for Reporting

In parallel with improved testing methodologies, the standardization of reporting practices is essential to enhance reproducibility and comparability across studies. Comprehensive reporting should include detailed characterization of material properties, environmental conditions, and experimental setup.

Material-related parameters such as degree of substitution, crosslinking density, porosity, and coating thickness should be clearly specified, as they directly influence swelling behavior, degradation rate, and diffusion characteristics. For polysaccharide-based systems, even minor variations in chemical modification can lead to significant changes in performance, making precise reporting critical.

Equally important is the description of environmental conditions, including soil type, pH, moisture content, ionic composition, and temperature. Given the strong sensitivity of polysaccharide matrices to these factors, their omission can lead to misinterpretation of results and hinder reproducibility.

Finally, experimental setup details such as liquid-to-solid ratio, agitation conditions, sampling intervals, and analytical methods must be explicitly reported. Standardizing these parameters will facilitate meaningful comparison between studies and support the development of unified evaluation criteria.

### 10.3. Proposed Evaluation Framework

To integrate these considerations into a coherent strategy, a stepwise evaluation framework is proposed for the assessment of polysaccharide-based fertilizer carriers.

The first stage involves comprehensive material characterization, including physicochemical properties, structural features, and modification details. This step establishes the baseline understanding required to interpret subsequent release behavior.

The second stage consists of conventional aqueous release testing, which remains useful for preliminary screening and comparison under controlled conditions. However, results obtained at this stage should be interpreted with caution and not considered fully representative of field performance.

The third stage introduces soil-relevant testing, incorporating either soil slurry systems, simulated soil solutions, or controlled moisture environments. This stage aims to capture the influence of environmental factors and coupled processes that govern nutrient release in realistic conditions. The proposed framework is also applicable to nano-nutrient-loaded and environmentally responsive polysaccharide gel systems, provided that formulation-specific parameters are incorporated into the relevant stages of evaluation [72,121,133]. For nano-nutrient-loaded gels, the material characterization stage should include nanoparticle size distribution, dispersion stability, aggregation tendency, nutrient dissolution behavior, and possible interactions between nano-nutrients, the polymer network, and soil components [72,133]. For environmentally responsive systems, the soil-relevant testing stage should include the specific triggering condition, such as pH, ionic strength, salinity, moisture fluctuation, temperature, or microbial activity [107,121]. In such cases, the framework does not change fundamentally; rather, it becomes more detailed by linking the selected stimulus or nano-nutrient property to the corresponding release mechanism and soil condition.

The final stage involves comparative analysis, where results from aqueous and soil-relevant testing are systematically evaluated to identify discrepancies, understand underlying mechanisms, and refine predictive models. This integrated approach allows for a more comprehensive assessment of material performance and supports the development of more reliable design principles.

**Figure 7:**
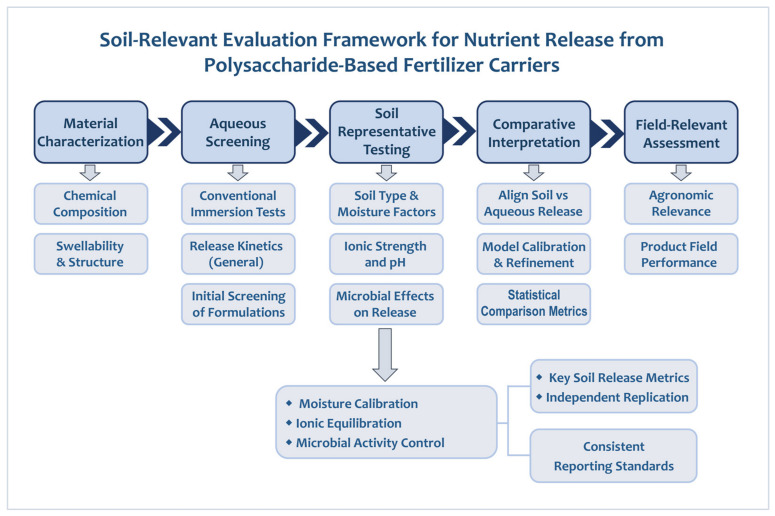
Proposed framework for soil-relevant evaluation of polysaccharide-based fertilizer systems. The approach integrates material characterization, conventional aqueous testing, soil-representative evaluation, and comparative analysis, and is supported by reported experimental strategies such as free-water/soil comparisons, soil-column testing, soil slurry systems, simulated soil solutions, and controlled wetting–drying evaluation.

By adopting such a structured framework, future studies can move beyond isolated and simplified testing practices toward a more holistic evaluation strategy. This progression is essential for advancing the field of polysaccharide-based fertilizer carriers and ensuring that laboratory innovations translate effectively into practical agricultural applications. The proposed framework is consistent with experimental strategies already reported in the controlled-release fertilizer literature [101,110,139]. For example, comparative release studies have evaluated nutrient release in free water, saturated sand, and sand at field capacity to demonstrate the strong influence of moisture availability and external transport resistance on release behavior [101,110]. Other studies have used soil incubation, soil-column testing, soil slurry systems, or simulated soil solutions to incorporate soil particles, ionic composition, pH effects, and physical confinement into release assessment [101,139]. Similarly, controlled wetting–drying approaches have been applied to better represent the intermittent hydration conditions experienced by fertilizer carriers under irrigation or rainfall cycles [107]. These examples indicate that soil-relevant evaluation does not replace conventional aqueous testing but should complement it through a progressive testing sequence, as summarized in Figure 7. A structured framework for soil-relevant evaluation and standardization of polysaccharide-based fertilizer carriers is presented in Figure 7.

## 11. Future Research Directions

The analysis presented in the preceding sections highlights a clear need to move beyond conventional evaluation practices toward more realistic, integrated, and predictive approaches for assessing polysaccharide-based fertilizer carriers. Future research should therefore focus not only on material development but also on advancing evaluation methodologies that can reliably capture the complexity of soil environments and their influence on nutrient release behavior.

One important direction involves the integration of experimental studies with mechanistic modeling approaches. Current models often rely on simplified assumptions derived from aqueous systems, which limits their applicability to soil conditions. Future efforts should aim to develop models that incorporate key soil-dependent parameters, including moisture variability, ionic interactions, and microbial activity. Such integrated frameworks would enable more accurate prediction of nutrient release kinetics and support rational material design.

Another critical area is the development of standardized, soil-representative testing protocols. Establishing unified guidelines that account for dynamic moisture conditions, realistic ionic compositions, and biological activity would significantly improve the comparability and reproducibility of experimental results. These protocols should move beyond static aqueous testing and incorporate conditions that better reflect field environments, thereby enhancing the relevance of laboratory findings.

Advancements in in situ and real-time monitoring techniques also offer significant potential. Emerging tools such as imaging methods, embedded sensors, and tracer-based approaches can provide direct insight into nutrient release and transport processes within soil systems. These techniques would enable continuous monitoring of release behavior under realistic conditions, offering a more detailed understanding of the interactions between material properties and environmental factors.

In addition, future research should adopt multi-scale evaluation strategies that bridge laboratory, mesocosm, and field studies. While laboratory experiments are essential for controlled investigations, intermediate-scale studies can provide valuable insights into system behavior under more complex conditions. Ultimately, validation at the field scale is necessary to confirm the practical effectiveness of proposed materials and evaluation methods.

A further important direction is the coupling of nutrient release studies with plant response and nutrient uptake efficiency. Traditional evaluation metrics, such as cumulative release percentage, do not fully capture the agronomic performance of fertilizer systems. Integrating plant-based assessments into evaluation frameworks would provide a more comprehensive understanding of how release behavior translates into actual agricultural benefits.

Finally, the design of advanced polysaccharide-based systems that respond to environmental stimuli represents a promising avenue for future development. Materials capable of adapting their release behavior in response to changes in soil pH, moisture, or ionic strength could offer improved efficiency and reduced nutrient loss. Such stimuli-responsive systems, when combined with soil-relevant evaluation methods, have the potential to significantly enhance the performance and sustainability of controlled-release fertilizers.

Addressing these research directions will be essential for advancing the field from simplified laboratory studies toward more realistic and application-oriented solutions, ultimately enabling the development of fertilizer systems that perform reliably under diverse soil conditions.

## 12. Conclusions

This review critically examined the evaluation of polysaccharide-based fertilizer carriers, with a particular focus on the widespread reliance on conventional aqueous testing methods. While such methods provide simplicity and experimental control, the analysis demonstrates that they systematically fail to capture the complex physicochemical and biological interactions governing nutrient release in soil environments. As a result, aqueous testing often leads to overestimation of release rates and misinterpretation of material performance.

The evidence presented across multiple studies confirms that soil conditions, including moisture variability, ionic composition, microbial activity, and structural confinement, fundamentally alter both the rate and mechanism of nutrient release. These factors introduce coupled processes that cannot be represented by simplified diffusion- or swelling-controlled models typically derived from aqueous systems. Consequently, laboratory-derived release data, when based solely on aqueous testing, have limited predictive value for real-world agricultural applications.

This work further identifies critical gaps in current evaluation practices, including the lack of standardized testing protocols, inconsistent reporting of experimental conditions, and insufficient integration of soil-relevant environments in performance assessment. These limitations hinder reproducibility, restrict cross-study comparison, and slow the development of reliable design principles for advanced fertilizer systems.

To address these challenges, a structured evaluation framework is proposed, emphasizing the progressive integration of soil-representative testing approaches, comprehensive reporting standards, and comparative analysis between aqueous and soil-based systems. Such an approach enables a more realistic and mechanistically informed assessment of material performance, bridging the gap between laboratory studies and field conditions.

The findings underscore the necessity of transitioning from simplified evaluation methodologies toward more holistic and soil-relevant strategies. This shift is essential not only for improving the accuracy of performance prediction but also for guiding the development of next-generation polysaccharide-based fertilizer carriers with enhanced efficiency and sustainability in practical agricultural applications.

## Figures and Tables

**Table 1:** Summary of representative studies on polysaccharide-based, polysaccharide-derived, and polysaccharide-containing hydrogel/coating systems for controlled-release fertilizers under soil-relevant conditions, highlighting composition, swelling behavior, water retention, and nutrient release kinetics.

Ref	Study ID	Modifier(s)	Modifier Type	Binder or Crosslinker	Release Time for 75% Release in Soil (d) (Estimated)	Swelling/Kinetics	Water Retention	Release Model
[43]	Gungula et al., 2021	Borax	Chemical	Borax	28.72	Scott’s 2nd order model, K and S∞ values reported	14.76% (with SRF) vs. 4.67% (control) after 4 days	Empirical cumulative release (%)
[44]	Sitthisuwannakul et al., 2023	Acrylamide and Montmorillonite	Chemical	MBA (crosslinker), KPS (initiator)	23.2	Fitted to zero-order, first-order, and Ritger–Peppas models	Not evaluated	Ritger–Peppas best fit (R^2^ = 0.997), indicating Fickian diffusion
[45]	Dong et al. 2021	Sodium Di-hydrogen Phosphate and Acrylamide	Chemical	Acrylamide (AM) + N,N′-methylene bisacrylamide (N-MBA)	48.28	Swelling ratio 80.2 g/g; BET surface area 0.7066 m^2^/g	48% weight loss over 13 days (vs. 70.5% for NS)	Not explicitly modeled
[46]	Wang et al., 2023	Liquefied starch polyol–modified bio-polyurethane and siloxane	Polysaccharide-derived hybrid bio-based coating	Liquefied starch polyol + castor oil–based polyurethane + siloxane	47.25	Surface pore sealing and hydrophobicity reduced swelling and water ingress	Not reported	Korsmeyer–Peppas + diffusion models
[47]	Lu et al., 2014	Xanthan gum, Acetic Anhydride and NaOH	Chemical	Trisodium trimetaphosphate (TSTP)	9.8	Water absorbency tested; swelling affected by XG/CMS ratio and TSTP %	41.1% (0%), 43.5% (1%), 46.6% (2%) soil water-holding with coated fertilizer	Not explicitly modeled
[48]	Li et al., 2024	Sodium alginate, Carboxymethyl Starch Sodium, Polydopamine	Chemical	Polydopamine	18.75	Swelling index tested	Improved significantly	Higuchi, Korsmeyer Peppas
[49]	Bilal Beig et al., 2020	Starch + PEG + Castor Oil + Diisocyanate	Hybrid (Chem + Natural)	Diisocyanate (MDI)	21	Fitted to 1st order + Korsmeyer-Peppas models	Improved soil water content and retention	First-order, Korsmeyer Peppas
[50]	Phansroy et al., 2024	Natural rubber, Sodium alginate	Chemical	Calcium chloride	29.16	IPN formation reduced swelling; less water uptake at higher NRL	Higher with more NRL in beads	Not fitted to mathematical model
[51]	Savitri et al., 2019	Chitosan, Acetic acid, Citric acid	Chemical	Citric acid (0.8% *w/w*)	5.25	Diffusion-controlled; Sample D had max swelling (25.1%)	Swelling higher in coated samples (up to 25.1%) than uncoated (7.8%)	Not modeled numerically; diffusion indicated
[52]	Tian et al., 2019	Diethylene glycol and Sulfuric acid	Chemical	Epoxy resin and isocyanate-based polyurethane	25.6	Swelling ratio decreased with higher epoxy content; no kinetic model reported	Not evaluated	Not modeled mathematically
[53]	Wei et al., 2020	Acrylamide and N,N′-methylene-bisacrylamide	Chemical	N,N-methylenebisacrylamide, Ammonium persulfate (initiator)	19.5	Burst + zero-order release at steady stage (10–17 days)	Not reported directly	Zero-order at intermediate stage
[54]	Akhter et al., 2021	Polyvinyl alcohol, Gelatin, Gum	Chemical	No crosslinkers mentioned; physical mixing with other polymers	5.35	Not reported	Soil water retention improved by coated NPK compared to uncoated	Not mathematically modeled
[55]	Chen et al., 2022	Sodium alginate	Chemical	Calcium chloride used for ionic crosslinking of alginate	0.34	Swelling ratio reduced with more CaCl_2_ crosslinking; higher alginate ratio reduced swelling	Not specifically measured, but swelling ratio indirectly indicates water uptake	Fitted to Ritger Peppas model (*n* < 0.43 indicates Fickian diffusion)
[47]	Lü et al., 2014	Poly(acrylic acid-co-acrylamide)	Chemical	N,N-methylenebisacrylamide as crosslinker; ammonium persulfate as initiator	14.16	Swelling capacity ~1170% in distilled water	Soil water-holding capacity increased by ~26–41%	Not explicitly modeled; data reported graphically
[56]	Lum et al., 2016	Boric Acid, Polyvinyl Alcohol	Chemical	Polyvinyl alcohol	3.75	Water uptake: 107–142% depending on urea content; swelling decreased with more urea	Indirectly implied via swelling data; not soil-tested	Release follows non-Fickian behavior; best fit with Korsmeyer Peppas model
[57]	Jyothi et al., 2018	Poly(acrylonitrile)	Chemical	Not explicitly mentioned	103.44	Slower release with higher % grafting	74.2–426.6%; decreased with higher grafting	Not explicitly modeled
[58]	Jin et al., 2012	Poly(acrylic acid-co-acrylamide)	Chemical	Crosslinked via radical copolymerization	37.5	Reduced swelling with increased crosslink density	8% lower transpiration rate than control; improved retention	Not specified
[59]	Perez et al., 2016	Chitosan and Sodium tripolyphosphate	Chemical	Sodium tripolyphosphate	11.3	Crosslinking time and matrix composition influenced swelling	Not directly studied	Empirical (cumulative release %)
[60]	Tian et al., 2021	Castor oil and Hexamethylene diisocyanate	Hybrid (Chem + Natural)	Hexamethylene diisocyanate	157.5	CO reduced porosity and modified film swelling	Not directly studied	Not mathematically modeled
[61]	Zhao et al., 2021	Bio-polyurethane and polyaryl polymethylene isocyanate	Hybrid (Chem + Natural)	PAPI (polyaryl polymethylene isocyanate)	112.5	SAPCU absorbed 120–160 g water/g; higher swelling with more SAP	Strong swelling capacity	Not specified
[62]	Sarkar et al., 2021	Polyvinyl alcohol and bentonite clay	Chemical	PVA (polyvinyl alcohol), bentonite clay	38.92	Slower water absorption at higher bentonite loadings	Lower porosity and water uptake	Korsmeyer–Peppas model
[63]	Swami et al., 2023	o-Phosphoric acid and Eggshell Nanoparticles	Chemical	Eggshell nanoparticles (ESN)	50	ESN improved elasticity and cracking control	Not specifically reported	Not explicitly modeled
[64]	Lu et al., 2025	Polyvinyl alcohol and Iron oxide nanoparticles	Chemical	Polyvinyl alcohol + Fe_2_O_3_ nanoparticles	26.8	Swelling enhanced with Fe_2_O_3_; structure more elastic	Significantly improved	Not explicitly modeled
[65]	Nakaramontri et al., 2022	Glycerol and Natural Rubber	Chemical	Blended with natural rubber and epoxidized NR	11.66	Water swelling capacity ~180%	Indirect via plant growth	Not explicitly modeled
[66]	Yan et al., 2021	Polyvinyl aclohol and boric acid	Chemical	Boric acid	131.32	Good swelling in salt solution	Excellent retention and pH control	Not explicitly modeled
[67]	Jia et al., 2020	Bio-polyurethane	Chemical	None reported	18.75	Swelling <10%; pseudo-second-order kinetics	Improved over control	Best fit: pseudo-second-order
[68]	Vudjung et al., 2018	Natural rubber, Gruteraldehyde, Sulfur, Wax	Chemical	Glutaraldehyde (GA), Sulphur (S)	18	Non-Fickian diffusion; *n* = 0.88 (water), 0.85 (soil); swelling 25.5% at 70/30	64.2% (for NR/St = 70/30)	Modified Peppas model
[69]	Beig et al., 2020	Polyvinyl alcohol, Parrafin wax, Molasses	Chemical	Molasses used as binding agent	33.75	Not specified	Improved water-holding capacity noted for treated soils (e.g., T3)	Not modeled
[70]	Ibrahim et al., 2020	Disodium borate decahydrate and urea	Chemical		16.1	Swelling capacity = 152–183 g/g; decreased with salt concentration	High water retention, especially in sandy soil	Not specified
[71]	Lu et al., 2023	Biochar, Hydrotalcite, Cellulose, CaH_6_O_9_P_2_, KCl	Chemical	No chemical binder; composite physically mixed	52.5	Water absorption capacity increased; retention higher in HT-rich blends	Improved water-holding capacity in sandy soil by 28.5–45.6%	Not specified
[72]	Priyanka et al., 2024	Montmorillonite nanoclay + Glycerol	Hybrid (Chem + Natural)	None reported	15	XRD confirms intercalation; TGA shows improved thermal stability	Enhanced significantly	Not specified
[73]	Hu et al., 2024	Carboxymethyl cellulose (CMC)	Hybrid (Chem + Natural)	Epichlorohydrin (ECH)	6.25	Maintained integrity after 72 h immersion; highly flexible	Not quantified	Not specified
[74]	Lv et al., 2024	Sodium Alginate	Hybrid (Chem + Natural)	Sodium alginate + Ca^2+^ ions	64.1	Water absorbency ~8.02 g/g; higher than native SA hydrogels	Excellent retention	First-order; non-Fickian
[75]	Phang et al.	Sodium Alginate and Calcium Chloride	Chemical	Calcium chloride (CaCl_2_) used as ionic crosslinker for alginate	22.5	Swelling ratio analyzed; higher CaCl_2_ reduced swelling	Not measured directly	Fitted to Ritger Peppas and Korsmeyer Peppas models
[76]	Zahid Majeed et al., 2016	Lignin and Disodium Tetraborate	Chemical	Urea	22.5	Reduced swelling with lignin; first-order kinetics	Not explicitly reported	First-order model
[77]	Zhao et al., 2025	Polyvinyl alcohol and Kaolinite	Chemical	None explicitly used	25	Reduced swelling due to kaolinite; improved mechanical integrity	Enhanced water retention	Not explicitly modeled

**Table 2:** Representative evidence and recurring observations underlying the laboratory–soil mismatch in evaluating nutrient release from polysaccharide-based fertilizer carriers.

Ref	System/Context	Testing Medium/Comparison	Main Observation	Implication for Evaluation
[9,140]	Polymer-coated fertilizers; comparative release experiments	Free water vs. saturated sand vs. sand at field capacity	Release was highest in aqueous systems and progressively reduced under more soil-like conditions	Conventional aqueous testing overestimates nutrient release relative to soil-relevant environments
[141,142]	Soil-like transport environment	Aqueous media vs. soil systems	Soil imposes external resistance through limited moisture, tortuous diffusion paths, and soil-particle interactions	Water-based tests miss external mass-transfer resistance present in real soils
[83,86,103]	Release kinetics of fertilizer systems	Aqueous testing vs. soil environments	Aqueous systems show shorter release duration and higher apparent release rate; soil systems show delayed and multi-stage release	Release duration and rate derived from water tests are not directly transferable to soil
[143]	Coated fertilizer nutrient release profiles	Medium-dependent release staging	Release may involve lag, constant-release, and declining phases, with stage duration depending on surrounding medium	Testing medium affects not just rate, but the shape and staging of release kinetics
[29,30,146]	Polysaccharide-based systems sensitive to soil chemistry and biology	Aqueous systems vs. ion-rich/biologically active soil	In soil, cation-mediated interactions reduce swelling while microbial activity introduces degradation-controlled pathways	Soil release is governed by coupled mechanisms absent from conventional aqueous tests
[13,147]	Polysaccharide-based hydrogels	Distilled water vs. realistic soil conditions	High swelling and rapid release in water become reduced swelling and slower release in soil because of ionic effects and confinement	Distilled-water testing can exaggerate hydrogel performance
[148]	Modified starch-based systems with hydrophobic grafting	Aqueous media vs. soil	Tunable release seen in water may not be maintained under soil conditions because of additional environmental interactions	Apparent formulation advantages in water require soil validation
[9,80]	Biodegradable coated fertilizer systems	Short-term aqueous tests vs. soil exposure	Materials appearing stable in short aqueous tests may degrade progressively in soil, accelerating later release	Water tests may miss degradation-assisted release occurring over time in soil
[13]	Practical/agronomic interpretation	Laboratory-derived release data vs. field performance	Overreliance on aqueous results risks overestimating fertilizer efficiency and misjudging release duration	Soil-representative testing is necessary for agronomic relevance
[149]	Predictive modeling based on simplified tests	Models fitted to aqueous data	Models assuming constant environment and single-mechanism control have limited applicability to soil systems	Predictive modeling should incorporate coupled soil processes

## Data Availability

The original contributions presented in this study are included in the article. Further inquiries can be directed to the corresponding author.
